# Characterising the performance of balanced memory networks

**DOI:** 10.1186/1471-2202-14-S1-P88

**Published:** 2013-07-08

**Authors:** Alex Metaxas, Reinoud Maex, Rod Adams, Volker Steuber, Neil Davey

**Affiliations:** 1 Science and Technology Research Institute, University of Hertfordshire, Hatfield, Hertfordshire, AL10 9AB, UK

## 

In previous work [1], we investigated the associative memory performance of networks of Izhikevich neurons with various synaptic plasticity regimes. However, the firing rates observed were far higher than those observed *in vivo*. In their recent Science paper [2], Vogels *et al*. describe a model of associative memory that exhibits biologically plausible firing rates, using a network of Integrate and Fire (IAF) neurons in which the inhibitory to excitatory synapses are plastic. Their self-organising learning rule provides a homeostatic function, leading to balanced excitation and inhibition. Further, by achieving a globally balanced state, the network displays asynchronous irregular dynamics. This sparse pattern of activity, which is present in cortical networks *in vivo*, enables rapid responses to small changes in the input [2].

The patterns are stored via a simplified form of one-shot Hebbian learning of synapses between excitatory neurons. The plastic inhibitory to excitatory synapses serve to balance the excitation in the memory assembly, mirroring the potentiated excitatory synapses and thereby allowing the stored pattern to be suppressed when not activated by external stimuli. This is a feature lacking in the ANN attractor networks we have studied previously [1], where the activation of a pattern causes the network to reach a fixed point.

In this work, we characterise the performance of the Vogels' network using a metric, *Effective Capacity*, adapted from [3]. Vogels uses a random architecture, with each neuron having a 0.02 probability of being connected to any other neuron. However, analyses of the connectivity of cortical networks have suggested that they may have non-random features [4]. Having measured the performance of the network with random connectivity, we measure the effect on performance of a small world architecture [3]. These results are contrasted with a similar study of connectivity patterns in (non-spiking) ANN networks [5].
